# Therapeutic effectiveness depends on propaedeutics

**DOI:** 10.1590/S1516-31802009000300001

**Published:** 2009-10-06

**Authors:** Álvaro Nagib Atallah

**Affiliations:** 1 Physician. Full professor and head of the Discipline of Emergency Medicine and Evidence-Based Medicine of Universidade Federal de São Paulo - Escola Paulista de Medicina (Unifesp-EPM). Director of the Brazilian Cochrane Center and Scientific Director of Associação Paulista de Medicina (APM). E-mail: atallahmbe@uol.com.br.

It is now well known and a matter of consensus both nationally and internationally that decision-making for medicine and healthcare should generally be based on good scientific evidence, particularly from clinical trials and systematic reviews with meta-analyses when possible.^1^

This conviction regarding the strategies to be followed has expanded especially because of the benefits accrued for patients, whose chances of success have increased and chances of harm have decreased, along with ethical, legal and economic benefits. The Brazilian Ministry of Health’s own experience has shown that the systematic reviews already conducted may have reduced the wastage of billions of reais per year on treatments of low efficacy that do not present advantages over traditional treatments, or that are even useless or harmful.^2^ All of this makes us believe that evidence-based decision-making is important for medical teaching and for healthcare managers.

The same is true for continuing education or, better, continuous professional development for physicians, since it is more efficient and easier to keep up to date with quality evidence than to have to be satisfied with information of questionable value in terms of practical results, from studies motivated by a variety of interests but with scientific deficiencies. Such studies cause wastage of financial resources, and this ends up subtracted from salaries and medical fees, and from the funding for policies of known efficacy, such as vaccination, cardiovascular disease prevention, food safety, hospital hygiene, etc.

However, everything said here so far starts from the principle that the diagnosis is correct and that the doubt lies only in what to do. But this is not so. Patients need to be heard calmly; complementary investigations need to be done carefully; physical examinations need to be detailed and not just directed; specialized physical examinations need to be competent and complete; differential diagnoses need to be based on wide-ranging medical knowledge; and approaches adopted need to have the good sense of aiming towards more benefit than harm for patients. In addition, patients need to be provided with information on the evidence regarding risks and benefits from each complementary and therapeutic diagnostic choice.

It is here that undergraduate education is decisive. Over recent decades, with the clear value placed on scientific production from teachers and the absence of tools for evaluating teaching at the bedside (and for evaluating patients), basic education for future professionals has come to have little worth in teaching careers within medical schools. Professionals whose activities consist almost exclusively of research, preferably at the laboratory bench, have these activities measured in terms of the number of papers published (on their merits), but basic education for clinical practice is tending to disappear (and I think that this is not an exaggeration). My question is: who is going to care for the health of our grandchildren and subsequent generations?

Despite all the blemishes of Brazilian medicine, its level is first-rate thanks to the teachers that we have had. However, it seems unlikely that it will be possible to replace them with the quality and vocation of the oldest of them. If for no other reason, this is because today, teaching activities have little influence on the clientele of our medical colleagues. Worse still, there is no academic or monetary incentive for our colleagues to improve their clinical propaedeutics and information, and thus to update their knowledge and their searches for and critical analyses on the evidence.

In any event, we started with teaching and I take the view that there is an urgent need to create separate career paths for clinical medical teachers, who should provide medical care and do clinical research when possible, and for teaching researchers. The latter would give emphasis to research and teaching, while clinical teachers would give emphasis to teaching and medical care (other proposals from readers would be welcome). The present setup is blatantly unfair to experts in clinical medicine, with apparent bias favoring the field of basic research, whereas the ideal would be to give equal value to clinical teaching and, deservedly, to basic research.

I take this opportunity to offer my congratulations to an expert and scholar of Brazilian medicine who has been an example of great benefit to our field and who has now reached the age of eighty years: Professor Dr. Adib Jatene. In addition, I ask for the blessing of some masters of inestimable value to the teaching of medical practice: Professor Domingos Delascio, Professor Osvaldo Luiz Ramos, Professor William Honsi, Professor Eurico de Jesus Zerbini, Professor Jairo Ramos, Professor Jorge A. Guimarães, Professor Octávio Ribeiro Ratto and Professor Mansur Gebara. We would like to ask readers to provide us with the names of their most esteemed teachers, for inclusion in a special issue of this journal. At the same time that we pay homage to them as examples to be followed, we (and readers) will present proposals for solutions to the problems mentioned here. The best way for us to defend medicine is for us to train physicians who are increasingly well prepared. We will always need better physicians and continuing education.
